# A simplified noncryogenic strategy to transport mesenchymal stem cells: Potential applications in cell therapy and regenerative medicine

**DOI:** 10.1016/j.gendis.2023.07.002

**Published:** 2023-08-24

**Authors:** Xiangyu Dong, Yannian Gou, Meichun Guo, Jiamin Zhong, Aohua Li, Ailing Hao, Wei Zeng, Rex C. Haydon, Hue H. Luu, Russell R. Reid, Tongchuan He, Yan Xu, Jiaming Fan

**Affiliations:** a Ministry of Education Key Laboratory of Diagnostic Medicine, And Department of Clinical Biochemistry, College of Laboratory Medicine, Chongqing Medical University, Chongqing 400016, China; b Molecular Oncology Laboratory, Department of Orthopaedic Surgery and Rehabilitation Medicine, The University of Chicago Medical Center, Chicago, IL 60637, USA; c Department of Interventional Neurology, The First Dongguan Affiliated Hospital, Guangdong Medical University, Dongguan, Guangdong 523475, China; d Laboratory of Craniofacial Suture Biology and Development, Department of Surgery Section of Plastic Surgery, The University of Chicago Medical Center, Chicago, IL 60637, USA; e Institute of Sports Medicine, Beijing Key Laboratory of Sports Injuries, Peking University Third Hospital, Beijing 100191, China

With the rapid advances in stem cell research and potential cell-based therapies, there is an urgent need to develop safe and reliable cell transport strategies. Except for autologous stem cell-based therapies, allogeneic stem cell therapies and *ex vivo* genetically engineered cell therapies would require safe, efficient, and reliable cell preservation and transport methods.^1^ Conventional cell transport methods include shipping live cells in medium-filled culture flasks at room temperature or ambient/hypothermic conditions (<35 °C) within several days, or expeditiously transporting cryogenically preserved cell vials in dry ice or liquid nitrogen,^2^ as it is commonly believed that hypothermic preservation benefits cell survival by reducing cellular metabolic rate and oxygen demand.^3^ While the former approach is convenient with variable outcomes, the latter is expensive, environmentally unfriendly, and technically challenging for long-distance transport. Here, using the mesenchymal stem cell (MSC) iMEFs as a model cell line,^4^ we developed a simplified, inexpensive, and noncryogenic method to transport cells, especially. By optimizing several crucial parameters including storage temperature, cell density, fetal bovine serum (FBS) concentration, and pH stability, we found that the cell viability remained 16%–18% when 2–10 × 10^5^ cells were stored in 1 mL of 2% FBS DMEM in a 1.5 mL Eppendorf tube at 4 °C–16 °C for up to 10 days. Furthermore, we demonstrated that the MSCs recovered from the above conditions maintained normal morphology, expressed stem cell markers, and retained osteogenic and adipogenic differentiation potential upon BMP9 stimulation *in vitro* and *in vivo*. Thus, our study identified an inexpensive, noncryogenic and reliable strategy for storage and transport of MSCs, potentially most mammalian cells.

To identify the optimal condition for noncryogenic cell transport, we first assessed cell viability at temperatures ranging from 4 °C to 37 °C. Using 2 × 10^5^ iMEFs suspended in 1 mL 10% FBS DMEM in 1.5 mL sterile Eppendorf tubes, we found the 4 °C group had more recovered living adherent iMEFs than the other 3 groups from days 3–10, while almost no cells survived at 37 °C from day 5 (Fig. 1A, a), which was confirmed by crystal violet staining (Fig. 1A, b). Quantitative flow cytometry analysis showed that viable cells accounted for 60.27%, 17.84%, 11.14%, and 7.46% of total iMEF cells recovered from 10-day storage at 4 °C, 16 °C, 23 °C, and 37 °C, respectively (Fig. 1A, c; Fig. S1). These results suggest that 4–16 °C, lower than ambient temperature, may be beneficial to cell survival for storage and transportation consuming more than 5 days. Thus, we chose 16 °C as the storage temperature for all further experiments.

**Figure 1:**
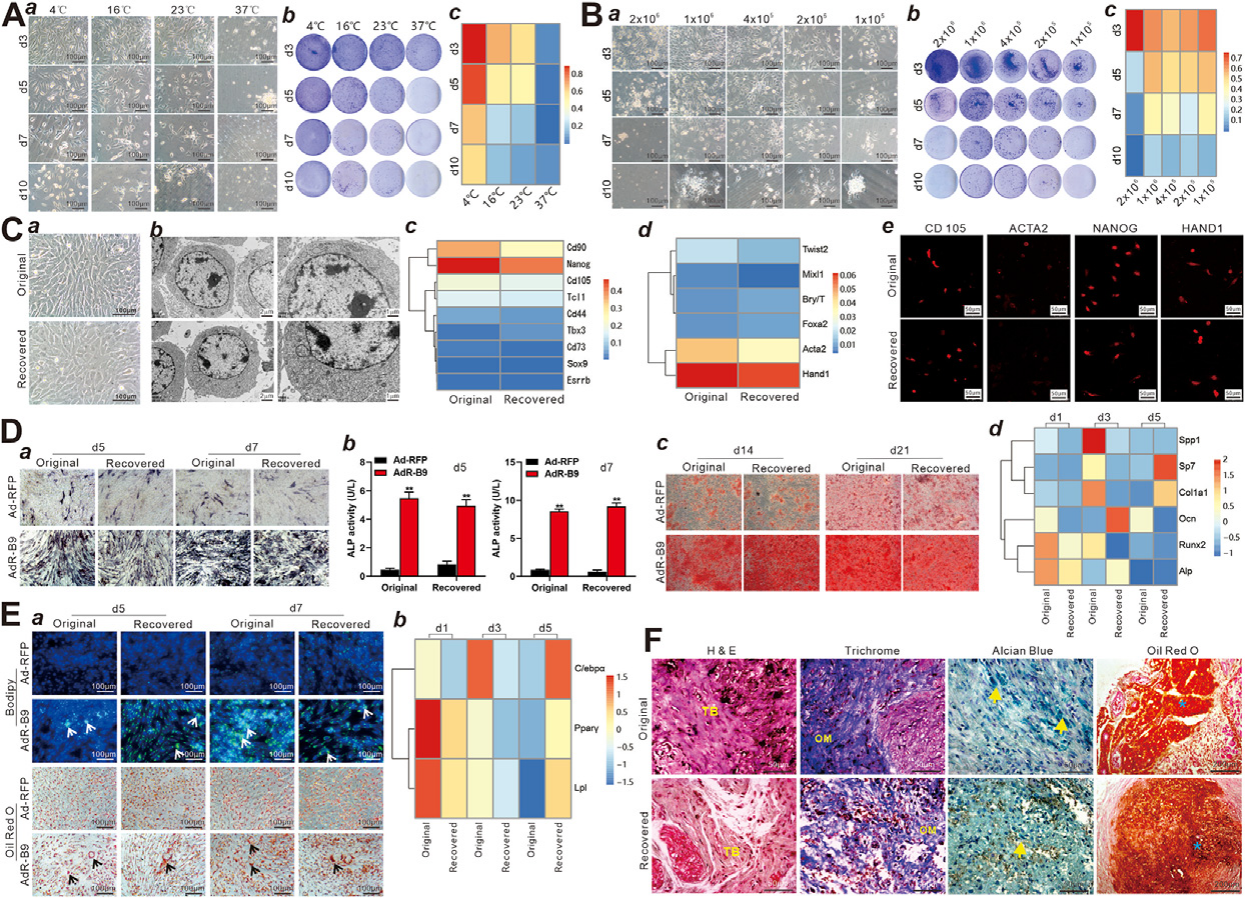
A simplified noncryogenic strategy to transport mesenchymal stem cells (MSCs). **(A)** The effect of storage/transport temperatures on the survival of iMEFs. 2 × 10^5^ iMEFs were suspended in 1 mL of 10% FBS DMEM in 1.5 mL sterile Eppendorf tubes, and kept at 4 °C, 16 °C, 23 °C, and 37 °C, separately, for 3, 5, 7, and 10 days. **(a)** The recovered iMEFs were plated and observed at 24 h under a bright field microscope (×200). **(b)** Crystal violet cell viability assay. The recovered iMEFs were plated in 35 mm cell culture dishes and stained with crystal violet at 24 h. Representative images are shown. **(c)** Flow cytometry-based apoptosis analysis. The original and recovered iMEFs were plated in 35 mm cell culture dishes, collected at 24 h, and stained with annexin V-FITC and propidium iodide (PI) for flow cytometry. More detailed flow cytometry results are presented in Figure S1. **(B)** The effect of cell densities on the survival of iMEFs. The iMEFs at 2 × 10^6^/mL, 1 × 10^6^/mL, 4 × 10^5^/mL, 2 × 10^5^/mL, and 1 × 10^5^/mL were separately suspended in 1 mL of 10% FBS DMEM in 1.5 mL sterile Eppendorf tubes and kept at 16 °C for 3, 5, 7, and 10 days. **(a)** Macrographic images of the recovered iMEFs. The recovered iMEFs were plated in 35 mm cell culture dishes and observed at 24 h under a bright field microscope (×200). Representative images are shown. **(b)** Crystal violet cell viability assay. The recovered iMEFs were plated in 35 mm cell culture dishes and stained with crystal violet at 24 h. Representative images are shown. (**c**) Flow cytometry-based apoptosis analysis. The original and recovered iMEFs were plated in 35 mm cell culture dishes, collected at 24 h, and stained with annexin V-FITC and PI for flow cytometry. More detailed flow cytometry results are presented in Figure S2. **(C)** The cell morphology and characteristics of marker gene expression of the recovered iMEFs. 4 × 10^5^ iMEFs were suspended in 1 mL of 2% FBS DMEM in 1.5 mL sterile Eppendorf tubes at 16 °C for 10 days. The original and recovered iMEFs were separately plated in 35 mm cell culture dishes and observed under a bright field microscope at 24 h (×100, ×200) **(a)** and transmission electron microscope (TEM) (×8000, ×15000) **(b)**. **(c,d)** TqPCR analysis. The original and recovered iMEFs were separately plated in 100 mm cell culture dishes and collected for RNA isolation at 24 h. TqPCR analysis was carried out to detect the expression of stemness markers **(c)** and mesoderm markers **(d)**. More detailed results are presented in Figure S6. **(e)** Immunofluorescence (IF) staining of MSC markers. The original and recovered iMEFs were separately seeded in chamber slides for 24 h and subsequently subjected to IF staining with antibodies against CD105, NANOG, ACTA2, and HAND1. The nuclei were counter-stained with DAPI (×400). Representative images are shown. More detailed results and negative controls are presented in Figure S7. **(D)** The osteogenic differentiation ability of the recovered iMEFs *in vitro*. 4 × 10^5^ iMEFs were suspended in 1 mL of 2% FBS DMEM in 1.5 mL sterile Eppendorf tubes and kept at 16 °C for 10 days. The original and recovered iMEFs were separately seeded in 24-well cell plates and infected with AdR-B9 or Ad-RFP for the following assays. Qualitative ALP staining **(a)** and quantitative ALP activity assay **(b)** were carried out on days 5 and 7. Representative images are shown in **(a)**. ^∗∗^*P* < 0.01, ^∗^*P* < 0.05, original iMEFs *vs*. the recovered iMEFs at the indicated time points. (**c**) Alizarin red staining was done on days 14 and 21. Representative images are shown. **(d)** TqPCR analysis was carried out to detect the expression of osteogenic markers on days 1, 3, and 5. Detailed results are shown in Figure S8. (**E**) The adipogenic differentiation ability of the recovered iMEFs *in vitro*. 4 × 10^5^ iMEFs were suspended in 1 mL of 2% FBS DMEM in 1.5 mL sterile Eppendorf tubes and kept at 16 °C for 10 days. The original and the recovered iMEFs were separately seeded in 24-well cell plates and infected with AdR-B9 or Ad-RFP. **(a)** Bodipy staining (top two rows) and oil red O staining (bottom two rows) were carried out on days 5 and 7. The lipid droplets were indicated by arrows. Representative images are shown. **(b)** TqPCR analysis was carried out to detect the expression of adipogenic markers on days 1, 3, and 5. Detailed results are shown in Figure S9. **(F)** The osteogenic and adipogenic differentiation potential of the recovered iMEFs *in vivo*. The original and recovered iMEFs were separately plated in 100 mm cell culture dishes and infected with AdR-B9 or Ad-RFP for 36 h. The infected cells were collected and subcutaneously injected into the ﬂanks of athymic nude mice. Subcutaneous masses were retrieved from AdR-B9 transduced cell groups after 5 weeks while no masses were retrieved in the Ad-RFP infected cell groups. The retrieved masses were subjected to hematoxylin–eosin staining, Masson's trichrome staining, alcian blue staining, and oil red O staining. Representative images are shown. TB, trabecular bone; OM, osteoid matrix. The cartilage was indicated by yellow arrows. The lipid droplets were indicated by asterisks.

We next determined the effect of cell densities on cell survival. Five cell densities (*i.e.*, 1 × 10^5^, 2 × 10^5^, 4 × 10^5^, 1 × 10^6^, 2 × 10^6^/mL) of iMEFs were suspended in 1 mL of 10% FBS DMEM in 1.5 mL Eppendorf tubes at 16 °C for 3, 5, 7, and 10 days. The recovered iMEFs were replated in 35 mm dishes. We found that while the number of live iMEFs in the 2 × 10^6^/mL group was higher than that in the other 4 groups on day 3, and the number in the 2 × 10^5^, 4 × 10^5^, or 1 × 10^6^/mL groups was more than that of the other 2 groups at day 7 and day 10 (Fig. 1B, a), which was confirmed by crystal violet staining (Fig. 1B, b). Flow cytometry analysis showed that, while 76.7% of iMEFs were viable in the 2 × 10^6^/mL group at day 3, which was significantly higher than that in other 4 groups, 16.7%, 11.7%, and 18.0% of iMEFs were viable in the 2 × 10^5^, 4 × 10^5^, and 1 × 10^6^/mL groups, respectively, after 10 days, although the total number of viable cells was the highest in the 1 × 10^6^ group (Figs. 1B, c; S2). These results indicate that more than 20% of cells, at least, can survive 10-day storage at 16 °C with cell density ranging from 2 × 10^5^/mL to 1 × 10^6^/mL.

We also tested the effect of FBS concentrations on cell survival by using 4 × 10^5^/mL iMEFs suspended in DMEM supplemented with 100%, 10%, 5%, or 2% FBS in 1.5 mL Eppendorf tubes at 4 °C for 3, 5, 7, and 10 days. Interestingly, at this storage temperature, no significant differences in cell morphology, cell viability, and survival rate were observed among different FBS concentration groups at the four time points (Fig. S3A–C), suggesting that the cells may survive at low-concentration (2% or 5%) or high-concentration (10% or 100%) FBS. We further analyzed whether pH changes caused by decreased CO_2_ concentrations during storage and transport would affect cell viability by supplying DMEM with HEPES to stabilize the medium pH. As shown in Figure S4, the addition of HEPES did not significantly improve overall cell viability, indicating that the iMEFs tolerated well in 2% FBS DMEM at 16 °C for up to 10 days (Fig. S4A–C). Additionally, we compared the survival rates between suspension storage and spheroid formation and did not observe any differences in survival rates at the four time points (Fig. S5A–D). Collectively, we arrived at an optimal and reliable storage/transport condition: 1 × 10^6^ iMEFs in 1 mL 2% FBS DMEM in 1.5 mL Eppendorf tube at 16 °C for up to 10 days.

Lastly, we investigated whether the storage/transport conditions impacted MSC morphology and functions using the above condition. By comparing with the original iMEFs, we did not find any cellular morphological changes in recovered iMEFs, except for slight swelling of mitochondria (Fig. 1C, a, b). TqPCR analysis with gene-specific primers (Table S1) revealed that MSC stemness markers and mesoderm markers were expressed at similar levels to that of the original iMEFs (Fig. 1C, c, d; Fig. S6A, B), which were confirmed by immunofluorescence staining (Fig. 1C, e; Fig. S7A, B). Using recombinant adenovirus AdR-B9 that overexpresses highly-osteogenic BMP9,^5^ we demonstrated that BMP9 induced similar levels of activities of the osteogenic marker alkaline phosphatase (ALP), mineralized nodule formation, and osteogenic gene expression in the original versus recovered iMEFs (Fig. 1D, a–d; Fig. S8). Furthermore, we showed that the recovered iMEFs retained similar adipogenic potential upon BMP9 stimulation to that of the original iMEFs (Fig. 1E, a, b; Fig. S9). By subcutaneously implanting the original and recovered iMEFs transduced by AdR-B9 (Fig. S10A, a, b), we found that BMP9 induced equally robust ectopic bone formation and adipogenesis in both groups (Fig. S10), which was confirmed by hematoxylin–eosin histologic analysis, Masson's trichrome staining, alcian blue staining, and oil red O staining (Fig. 1F). Collectively, these findings demonstrate that the recovered iMEFs retained multipotent differentiation potential of MSCs.

In conclusion, using iMEFs as a model progenitor line, we optimized several crucial parameters including storage temperature, cell density, FBS concentration, and pH stability, and demonstrated that the cell viability remained >20% when 2–10 × 10^5^ cells/mL in 2% FBS DMEM were stored at 4–16 °C for up to 10 days. MSCs recovered from the above conditions maintained normal morphology, expressed stem cell markers, and retained osteogenic and adipogenic differentiation potential upon BMP9 stimulation. It is conceivable that the use of a wet ice package is a practical means to keep the temperature at 4–16 °C for long-distance shipment of mammalian cells.

## Author contributions

JF, XD, and TCH conceived and designed the study. XD, YG, MG, and JZ performed the experiments and collected data. XD and JF performed the statistical analysis. AL, AH, and WZ participated in experiments, provided essential experimental materials, and assisted in qPCR data analysis and interpretations. TCH, JF, XD, RCH, HHL, RRR, and YX drafted and revised the manuscript. All authors reviewed and approved the final manuscript.

## Conflict of interests

The authors declare no competing conflict of interests.

## Funding

The reported work was supported in part by research grants from the 10.13039/501100001809Natural Science Foundation of China (No. 82102696 to JF), the 2019 Funding for Postdoctoral Research (10.13039/501100011786Chongqing Human Resources and Social Security Bureau No. 298 to JF), and the 10.13039/100000002National Institutes of Health (No. CA226303 to TCH, DE030480 to RRR). TCH was also supported by the Mabel Green Myers Research Endowment Fund and The University of Chicago Orthopaedics Alumni Fund. Funding sources were not involved in the study design; in the collection, analysis, and interpretation of data; in the writing of the report; and in the decision to submit the paper for publication.

## Data availability

All datasets generated for this study are included in the manuscript and/or the supplementary data. Any further inquiries about data and resource availability can be directed to the corresponding authors.
