# Supplementary material for: A simplified noncryogenic strategy to transport mesenchymal stem cells: Potential applications in cell therapy and regenerative medicine
Source: Genes Dis. 2023 Aug 24;11(3):101073. doi: 10.1016/j.gendis.2023.07.002 (PMC10808911; doi:10.1016/j.gendis.2023.07.002)
Supplement: Multimedia component 1 [file mmc1.docx]

**SUPPLEMENTAL MATERIALS**

**Materials and Method**

**Cell culture and chemicals**

The immortalized mouse embryonic fibroblasts (iMEFs) and HEK-293 derivatives 293pTP cells were previously described ^1-4^. All cells were cultured in DMEM supplemented with 10% fetal bovine serum (Lonsera, Cat: S711-0015, Uruguay) containing 100 units of penicillin and 100 µg/mL of streptomycin in 5% CO_2_ incubators at 37°C as described ^5-8^. For spheroid culture formation, exponentially growing iMEFs were suspended and cultured in ultra-low attachment cell culture plates with medium change every 2 days. When the size of spheroids reached 150-250 mm in diameter, the spheroids were collected for experiments as described ^9-12^. The cell morphology was analyzed under a bright field microscope. Unless otherwise stated, all chemicals were purchased from Sigma-Aldrich (St Louis, MO, USA), Thermo Fisher Scientific (Waltham, MA, USA), or Solarbio (Beijing, China).

**Crystal violet cell viability assay**

The crystal violet cell viability assay was performed as previously described^13;14^. Brieﬂy, the original iMEFs and the recovered iMEFs at indicated time points were seeded in 35 mm cell culture dishes. At 24 h After plating, adherent cells were gently washed with PBS and stained with 0.5% crystal violet/formalin solution for 5 min. The stained cells were washed with tape water and air-dried for scanning. Each assay condition was done in triplicate.

**Apoptosis analysis using flow cytometry**

The original and recovered iMEF cells were stained with annexin V-FITC and Propidium Iodide (PI) in darkness for 20 minutes at RT, followed by analyzed by flow cytometry (Beckman Coulter, USA) as described ^8^.

**Transmission electron microscope (TEM) analysis**

TEM analysis was performed as previously described ^15^. Brieﬂy, 2 × 10^6^ iMEF cells were collected and fixed with 2.5% glutaraldehyde at 4℃, washed with 0.1M sodium cacodylate, fixed with 4% osmium tetroxide, followed by serial dehydration in ascending concentrations of acetone. 90nm-thick sections were stained with lead citrate/uranyl acetate, and analyzed under a transmission electron microscope (Hitachi 7500, Japan, magnification × 8000 or × 15000).

**Total RNA extraction and touchdown-quantitative real-time PCR (TqPCR)**

Total RNA was extracted from iMEFs by using TRIZOL Reagent (Invitrogen, China) as described ^16-18^. Brieﬂy, total RNA was subjected to reverse transcription with hexamer and M-MuLV reverse transcriptase (New England Biolabs, Ipswich, MA). The cDNA products were used as PCR templates. Gene-specific qPCR primers were designed by using Primer3 program and were shown in the **Table S1**. TqPCR was carried out as described ^19^ by using 2 × SYBR Green qPCR Master Mix (Bimake, Shanghai, China) on the CFX-Connect unit (Bio-Rad Laboratories, Hercules, CA). All TqPCR reactions were done in triplicate. *Gapdh* was used as a reference gene. Quantification of gene expression was calculated by using the 2^-ΔΔCq^ method as described ^20^.

**Construction and amplification of recombinant adenoviral vectors AdR-B9 and Ad-RFP**

The AdEasy technology was used to generated recombinant adenoviral vectors as described ^21-23^. The construction of AdR-B9 overexpressing the mouse Bmp9 was described previously ^24^. Ad-RFP was used as a control virus. Adenoviruses were amplified in 293pTP cells. Fluorescence signals were documented under a fluorescence microscope at 48h after infection. Polybrene (8µg/mL) was added to enhance infection efficiency as described ^25^.

**Immunofluorescence (IF) staining**

The IF staining was carried out as reported previously ^13;14^. Briefly, exponentially growing iMEFs were seeded in chamber slides were fixed with 4% paraformaldehyde for 30 min at RT, treated with 0.5% Triton X-100 for 10 min, and blocked with 5% bovine serum albumin (BSA) for 30min at RT, followed by incubating with primary antibodies against CD105 (1:100 dilution; Bioworld; Cat# MB63153), NANOG (1:100 dilution; proteintect; Cat# 14295-1-AP), ACTA2 (1:50 dilution; Bimake; Cat# A5550) and HAND1 (1:100 dilution; bioworld; Cat# MB63487) overnight. After being washed, the cells were incubated with DyLight 594, goat anti-mouse IgG (1:200 dilution; Abbkine; Cat# A23410) or goat anti-rabbit IgG (1:200 dilution; Bioss; Cat# bs-0295G-APC) for 2 h at RT. Cell nuclei were counterstained with DAPI (10 μg/mL) for 10 minutes at RT. Minus primary antibody or control IgG was used as negative controls which shown in **Fig. S7**. Fluorescent images were obtained with a laser confocal microscope (Leica TCS SP8, magnification × 400). Each assay condition was done in triplicate.

**Alkaline phosphatase (ALP) activity**

ALP activity was assessed qualitatively with histochemical staining as described previously ^15;26;27^. Briefly, AdR-B9 or Ad-RFP was used to infect subconfluent iMEFs, at the indicated time points cells were fixed with glutaraldehyde and stained with a mixture of naphthol AS-MX phosphate and Fast Blue BB salt. The stains were washed with PBS and recorded. Staining results were recorded under a bright field microscope (magnification, × 100). ALP activity was also quantitatively assessed with the Nativelysis Buffer (Solarbio, Cat#R0030) and the AKP/ALP activity test kit (Solarbio, Cat#BC2145) at indicated time points as described ^15^. Each assay condition was done in triplicate.

**Alizarin Red S staining**

The Alizarin Red S staining was conducted as described ^27-29^. Briefly, exponentially growing iMEFs were seeded in 24-well plates and infected with AdR-B9 or Ad-RFP. The infected cells were cultured in complete DMEM with dexamethasone (100 nM), ascorbic acid (50 μM) and β-glycerophosphate (10 mM). At the endpoints of assays, the mineralized matrix nodules were stained for calcium precipitation by Alizarin Red S staining. The staining of calcium mineral nodules was air-dried for image scanning, and recorded under a bright field microscope (magnification, × 100). Each assay condition was done in triplicate.

**Bodipy 493/503 lipid droplet staining**

Bodipy 493/503-based fluorescence detection of lipid droplets was carried out as previously reported ^30;31^. Briefly, subconfluent cells infected with AdR-B9 or Ad-RFP were seeded in 24 well culture plates, fixed with 4% paraformaldehyde for 30 min and stained with 2 µmol/L BODIPY493/503 (4,4-Difluoro-1,3,5,7,8-Pentamethyl-4-Bora-3a,4a-Diaza-s-Indacene) for 15 min at indicated time, followed by PBS washes. The staining images were obtained under a fluorescence microscope. Each assay condition was done in triplicate.

**Oil Red O staining assay**

Oil Red O staining was carried out as described ^15;30;31^. Briefly, subconfluent iMEFs were seeded in 24-well cell culture plates and infected with AdR-B9 or Ad-RFP. At the indicated time points, cells were fixed with 4% paraformaldehyde for 30min, washed with PBS and subjected to Oil Red O staining. For the retrieved bone masses, the bone masses were fixed, decalcified, and subjected to frozen sectioning, followed by Oil Red O staining. Staining results were examined under a bright field microscope. Each assay condition was done in triplicate.

**Subcutaneous iMEFs implantation**

The use and care of experimental animals was approved by the Research Ethics and Regulations Committee of Chongqing Medical University, Chongqing, China. All experimental procedures followed the approved guidelines. Athymic nude mice were obtained from and housed in the Experimental Animal Research Center of Chongqing Medical University. The subcutaneous iMEF cells implantation was carried out as previously described ^7;32;33^. Briefly, iMEF cells were infected with AdR-B9 for 24 h, collected and resuspended in sterile PBS for subcutaneous injection into the ﬂanks of athymic nude mice (5–6-week-old, male, 5 × 10^6^ cells/injection, 4 injections per mouse, and 3 mice in total). At 5 weeks after injection, mice were sacrificed, and the subcutaneous masses were retrieved and subjected to paraffin embedding and histologic and IHC staining.

**Histological staining and evaluation**

Retrieved masses were fixed, decalcified in 10% formalin, embedded for paraffin or frozen sections. Serial sections were subjected to H & E staining, Masson’s trichrome staining (Masson's Trichrome Stain Kit, G1340, Solarbio, China), Alcian Blue staining (Alcian Blue Cartilage Stain Kit, pH 1.0, G2541, Solarbio, China) and Oil Red O Staining as described. Staining results were recorded under a bright field microscope (magnification, × 100 or × 400). Each assay condition was done in triplicate.

**Statistical analysis**

All experiments performed at least three times and/or repeated in three independent batches. Data were analyzed using GraphPad Prism 8 and presented as mean ± standard deviation (SD). Statistical significance was confirmed by one-way analysis of variance and the student’s *t*-test for the comparisons between groups. A value of *P* < 0.05 was considered statistically significant.

| **Table S1. List of TqPCR Primers** | | | |
| --- | --- | --- | --- |
| Gene | Forward primer | Reverse Primer | Accession No. |
| Mouse *GAPDH* | ATGCCATCACTGCCACCC | GCCAGTGAGCTTCCCGTT | NM_001289726.1 |
| Mouse *Bmp9* | CCGCTGCAGAACTGGGAA | GTGGCTCCGCTCTGGTTT | NM_019506.4 |
| Mouse *Cd44* | GGCAATGGGACGGTGGAA | CTGGTCCGGGGTCTCTGA | NM_001177787.1 |
| Mouse *Cd90* | AACCTTCGCCTGGACTGC | CCAAGGGTGCCTGAGAGC | NM_009382.3 |
| Mouse *Sox9* | TGACCGACGAGCAGGAGA | GGGTGTTCTCCGTGTCCG | NM_011448.4 |
| Mouse *Esrrb* | CTGGACTCGCCGCCTATG | AATGTCCCCGCACACGAG | NM_001159500.1 |
| Mouse *Cd105* | CCTGCTCACAGCTGCACT | CCCGATGCTGTGGTTGGT | NM_001146348.1 |
| Mouse *Nanog* | CCTCTCCTCGCCCTTCCT | CTCCTCCTCAGGGCCCTT | NM_001289828.1 |
| Mouse *Tbx3* | GATGAGCGCAGGAGTCCC | TGAAGAACTGCTGGCCCG | NM_011535.3 |
| Mouse *Cd73* | ACTCAGACGTGCCGCTTC | GCAGCCAGGTTCTCCCAG | NM_011851.4 |
| Mouse *Tcl1* | GAGACACCTGCACACCCC | TGGCGCAAGATCACCTGG | NM_001289468.1 |
| Mouse *Acta2* | CAGGCTGTGCTGTCCCTC | CAGCCAAGTCCAGACGCA | NM_007392.3 |
| Mouse *Bry/T* | TGGAGGAGAGCGAGCTGT | GGTTGTCAGCCGTCACGA | NM_009309.2 |
| Mouse *Hand1* | GACGTGCTGGCCAAGGAT | AGCTTTCGGGCTGCTGAG | NM_008213.2 |
| Mouse *Foxa2* | CCAACAAGATGCTGACGCTG | TTCTCGAACATGTTGCCCGA | NM_001291065.1 |
| Mouse *Mixl1* | CCAAACCGCGACTCCAGT | GCTCCCGCAAGTGGATGT | NM_013729.3 |
| Mouse *Twist2* | CGCTCCCCTCTGACAAGC | GTGAGCCACGTAGCTGCA | NM_007855.3 |
| Mouse *Runx2* | CCGGTCTCCTTCCAGGAT | GGGAACTGCTGTGGCTTC | NM_001271631.1 |
| Mouse *Sp7* | GGGAGCAGAGTGCCAAGA | TACTCCTGGCGCATAGGG | NM_001348205.1 |
| Mouse *Alp* | CCCCATGTGATGGCGTAT | CGGTAGGGAGAGCACAGC | NM_007431.3 |
| Mouse *Spp1* | CTCCTCCCTCCCGGTGAA | GCATTCTGTGGCGCAAGG | NM_001204203.1 |
| Mouse *Ocn* | CCTTCATGTCCAAGCAGGA | GGCGGTCTTCAAGCCATAC | NM_001032298.3 |
| Mouse *Col1a1* | GAGCGGAGAGTACTGGATCG | GCTTCTTTTCCTTGGGGTTC | NM_007742.4 |
| Mouse *Pparγ* | TGCCTTGCTGTGGGGATG | CCAGGGCTCGCAGATCAG | NM_011146.4 |
| Mouse *C/ebpa* | CCCCGATGAGCAGTCACC | ACTCGTCGTTGAAGGCGG | NM_001287514.1 |
| Mouse *Lpl* | GGCCGCCCTGTACAAGAG | GGCTCCAAGGCTGTACCC | NM_008509.2 |


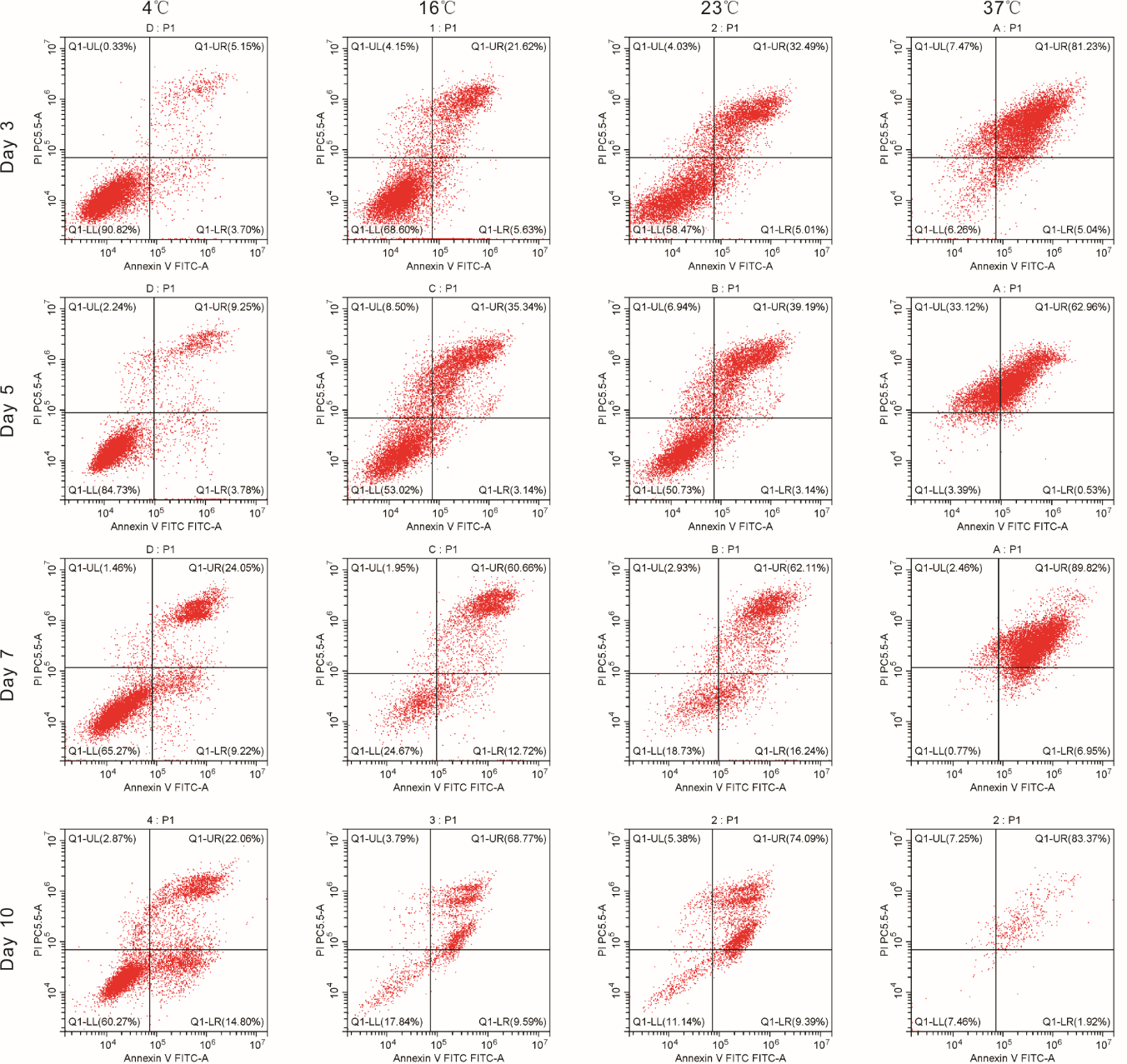


**Figure S1. The effect of storage/transport temperatures on the survival of iMEFs.** 2 × 10^5^ iMEFs were suspended in 1 mL of 10% FBS DMEM in 1.5 mL sterile EP tubes, and kept at 4℃, 16℃, 23℃ and 37℃, respectively. The original and recovered iMEFs were plated in 35 mm cell culture dishes, collected at 24 h, and stained with Annexin V-FITC and PI for flow cytometry. Representative images are shown. The results are presented as a heatmap in **Fig. 1A, panel *c***.


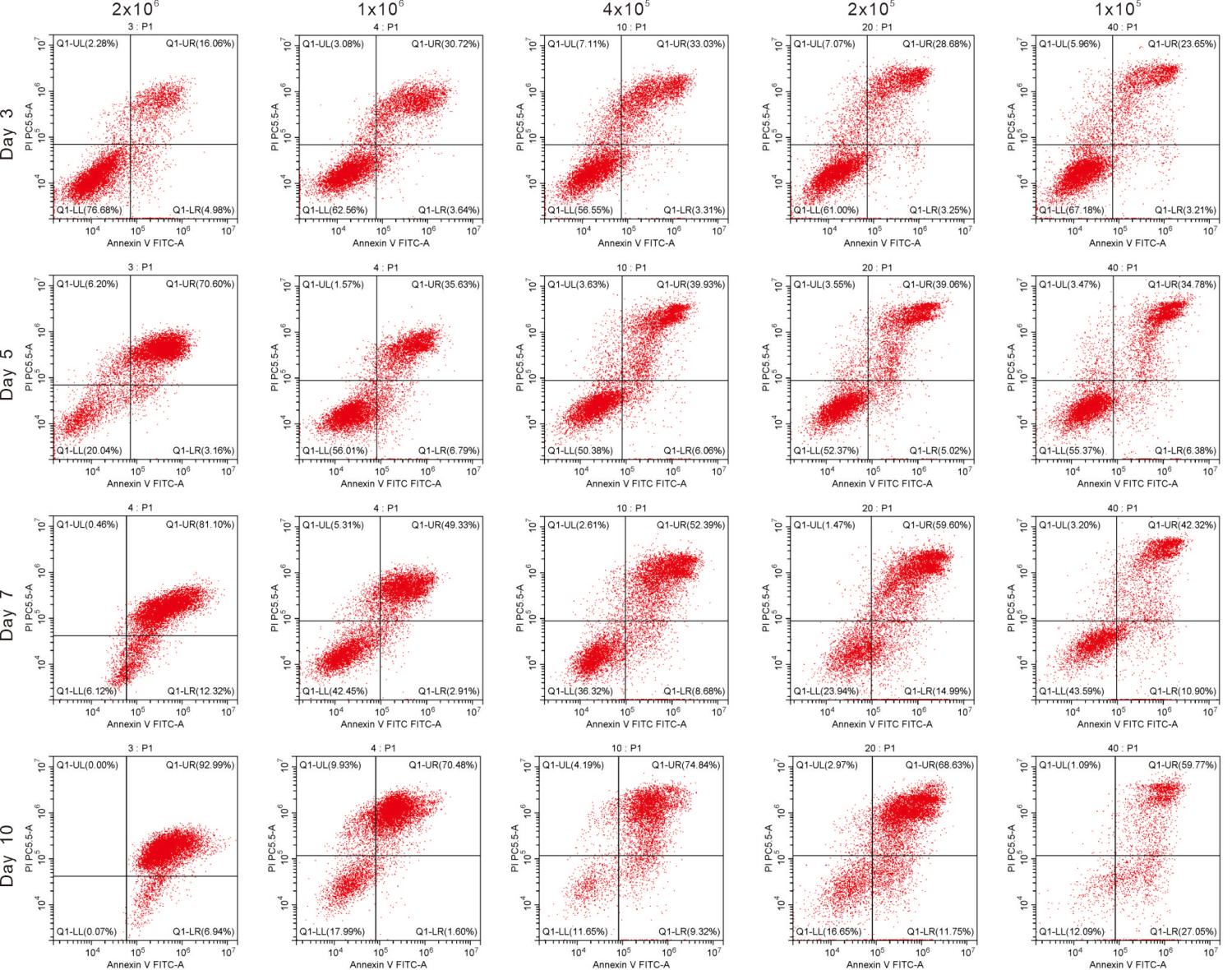


**Figure S2. The effect of cell densities on the survival of iMEFs.** 2 × 10^6^, 1 × 10^6^, 4 × 10^5^, 2 × 10^5^ and 1 × 10^5^ iMEFs were suspended in 1 mL of 10% FBS DMEM in 1.5 mL sterile Eppendorf tubes, and kept at 16℃ for 3, 5, 7 and 10 days. The original and recovered iMEFs were plated in 35 mm cell culture dishes, collected at 24 h, and stained with Annexin V-FITC and PI for flow cytometry. Representative images are shown. The results are presented as a heatmap in **Fig. 1B, panel *c***.


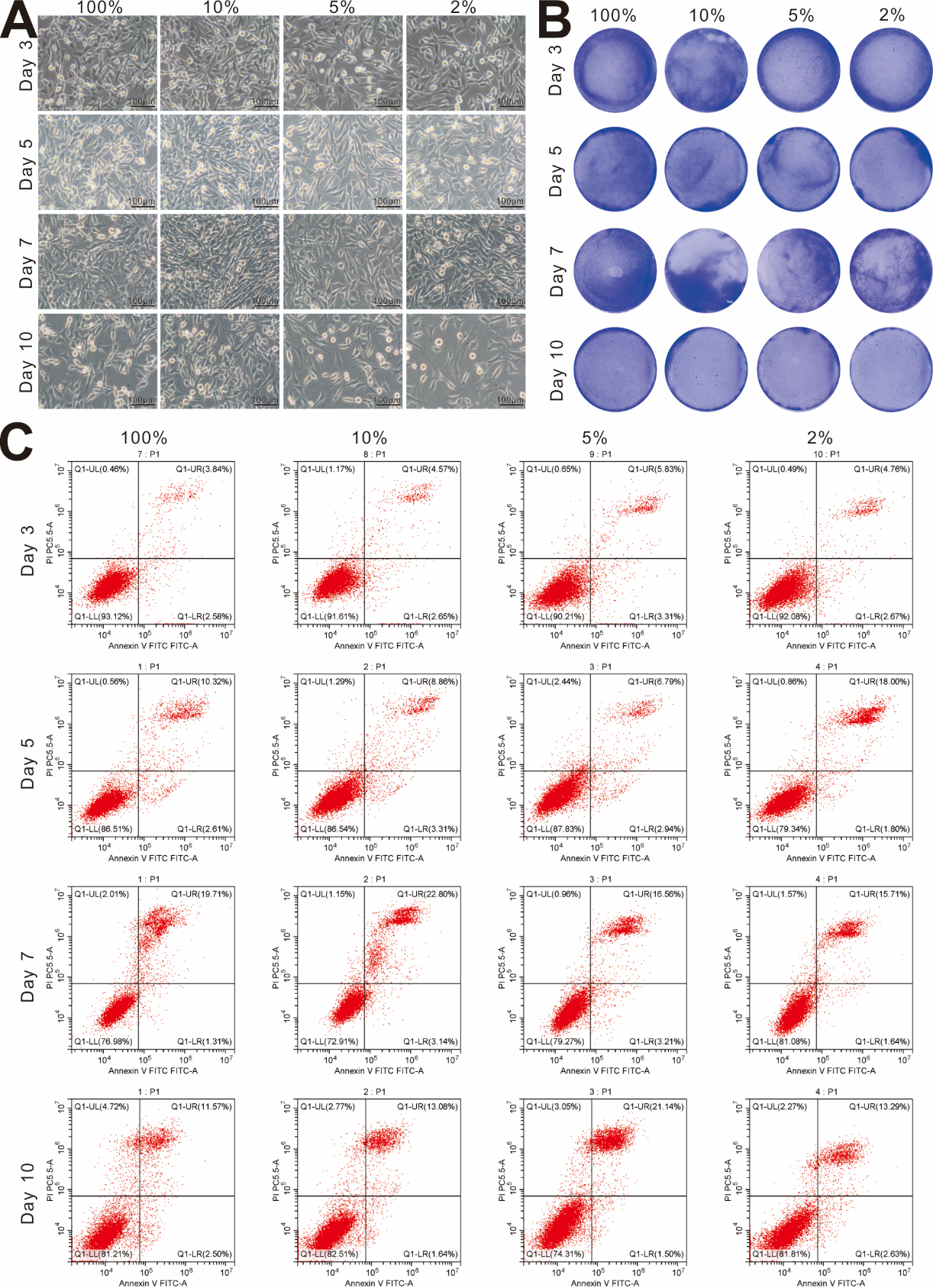


**Figure S3. The effect of fetal bovine serum (FBS) concentrations on the survival of iMEFs.** 4 × 10^5^ iMEFs were suspended in 1 mL of DMEM supplemented with 100%, 10%, 5% and 2% FBS in 1.5 mL sterile Eppendorf tubes and kept at 4℃. **(A)** The cell morphology of the recovered iMEFs was recorded at days 3, 5, 7 and 10 of storage, respectively (× 200). **(B)** Crystal violet cell viability assay was used to assess the cell viability and proliferation of the recovered iMEFs at days 3, 5, 7 and 10, respectively. (**C**) Flow cytometry-based apoptosis analysis of recovered iMEFs was conducted at days 3, 5, 7 and 10, respectively. Representative images are shown.


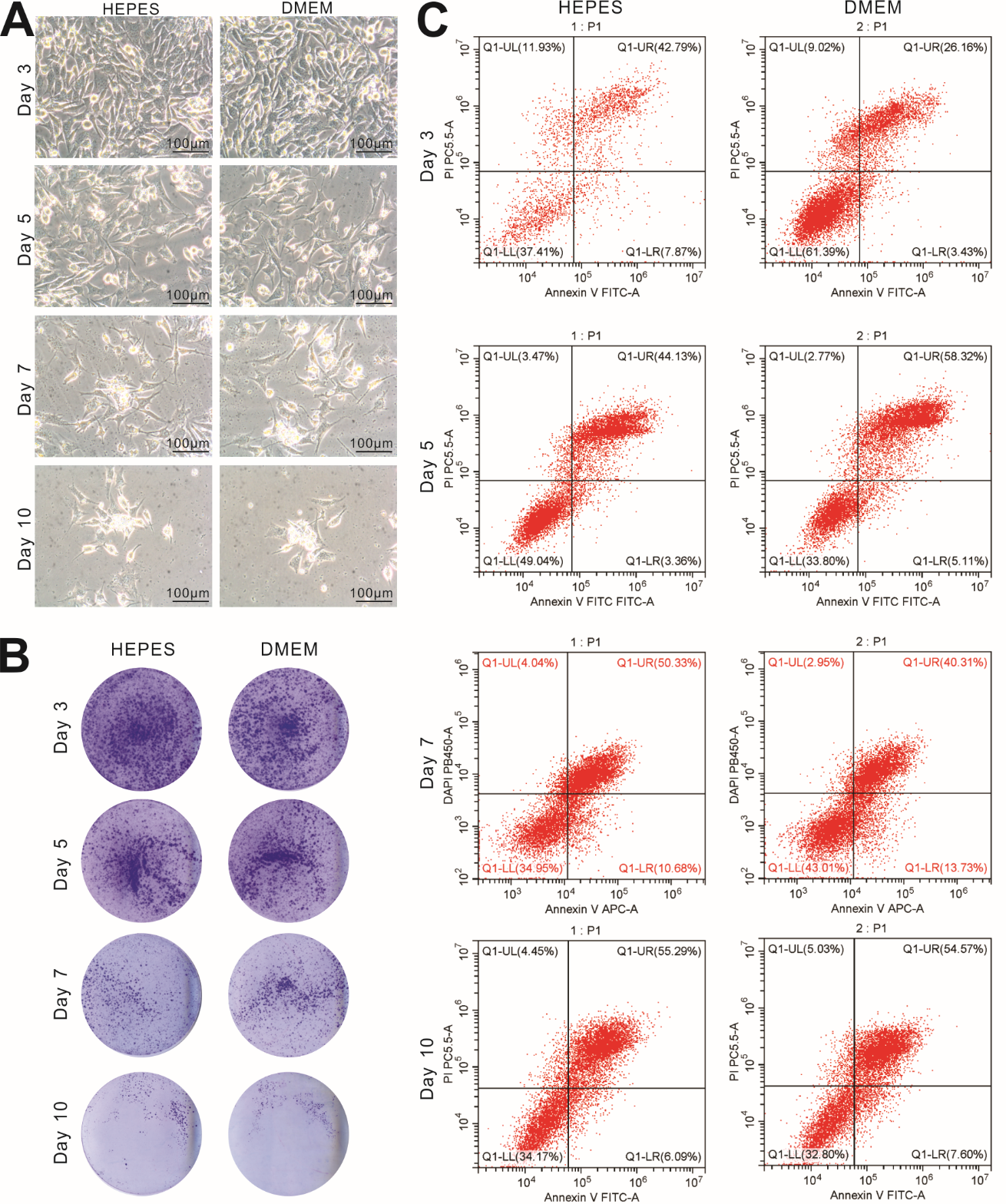


**Figure S4**. **The effect of pH buffers on the survival of iMEFs.** 4 × 10^5^ iMEFs were suspended in 1mL of 2% FBS DMEM supplemented with HEPES or without HEPES (DMEM) in 1.5 mL sterile Eppendorf tubes and stored at 16℃. **(A)** The cell morphology of the recovered iMEFs was recorded at days 3, 5, 7 and 10 of storage, respectively (× 200). **(B)** Crystal violet cell viability assay was used to assess the cell viability and proliferation of the recovered iMEFs at days 3, 5, 7 and 10, respectively. **(C)** Flow cytometry-based apoptosis analysis of recovered iMEFs was conducted at days 3, 5, 7 and 10, respectively. Representative results are shown.


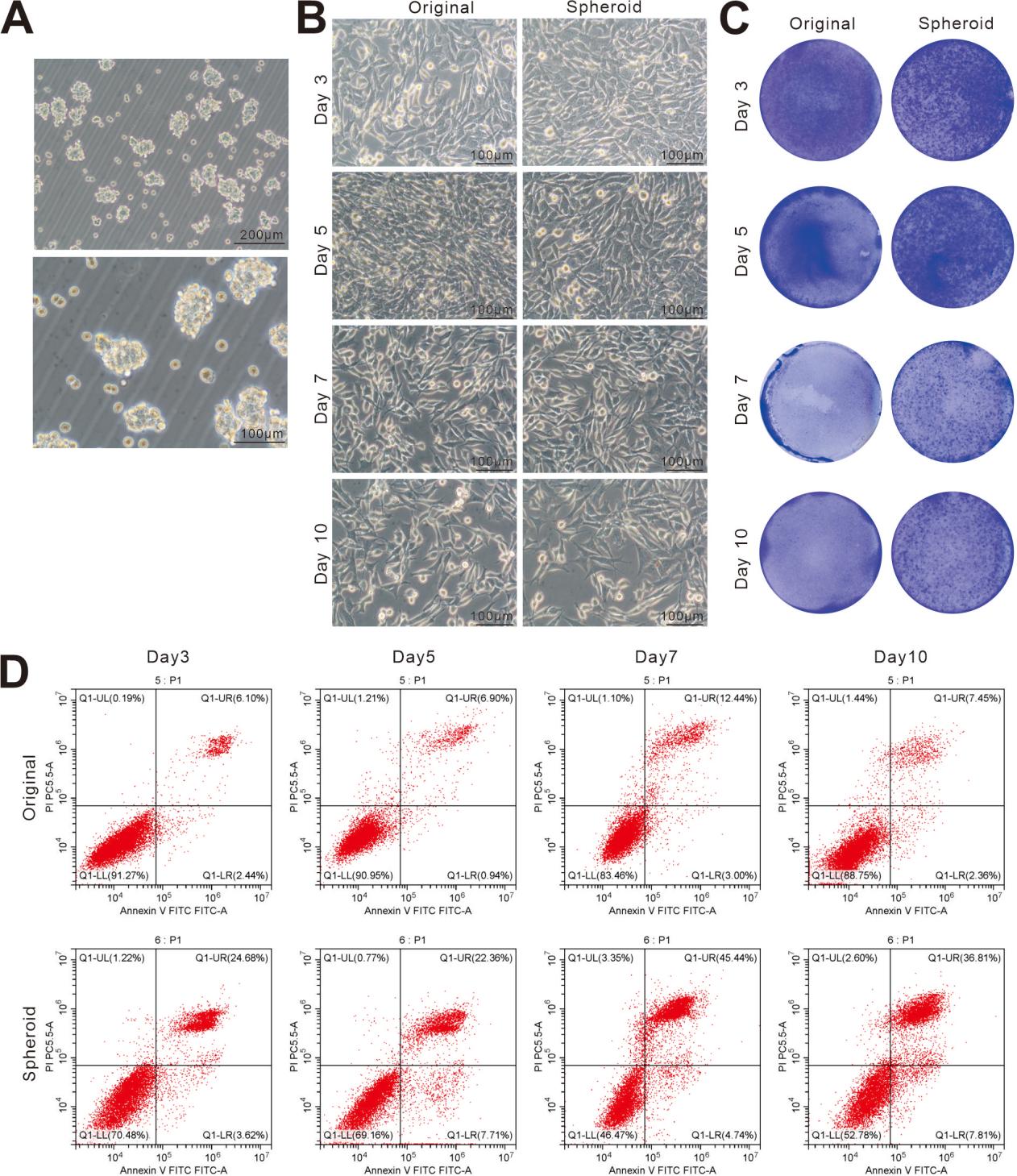


**Figure S5. The effect of spheroid formation on the survival of iMEFs.** 4 × 10^5^ iMEFs were suspended in 1 mL of 10% FBS DMEM in 1.5 mL sterile Eppendorf tubes, and stored at 4℃ for spheroid formations. **(A)** The spheroid formation was observed under light microscope (× 100, × 200). **(B)** Macrographic images of the iMEFs recovered from spheroids were recorded at days 3, 5, 7 and 10, respectively, under light microscope (× 200). **(C)** Crystal violet cell viability assay was used to assess the cell viability and proliferation of the iMEFs recovered from spheroids at days 3, 5, 7 and 10, respectively. **(D)** Flow cytometry-based apoptosis analysis of the iMEFs recovered from spheroids was conducted at days 3, 5, 7 and 10, respectively. Representative results are shown.


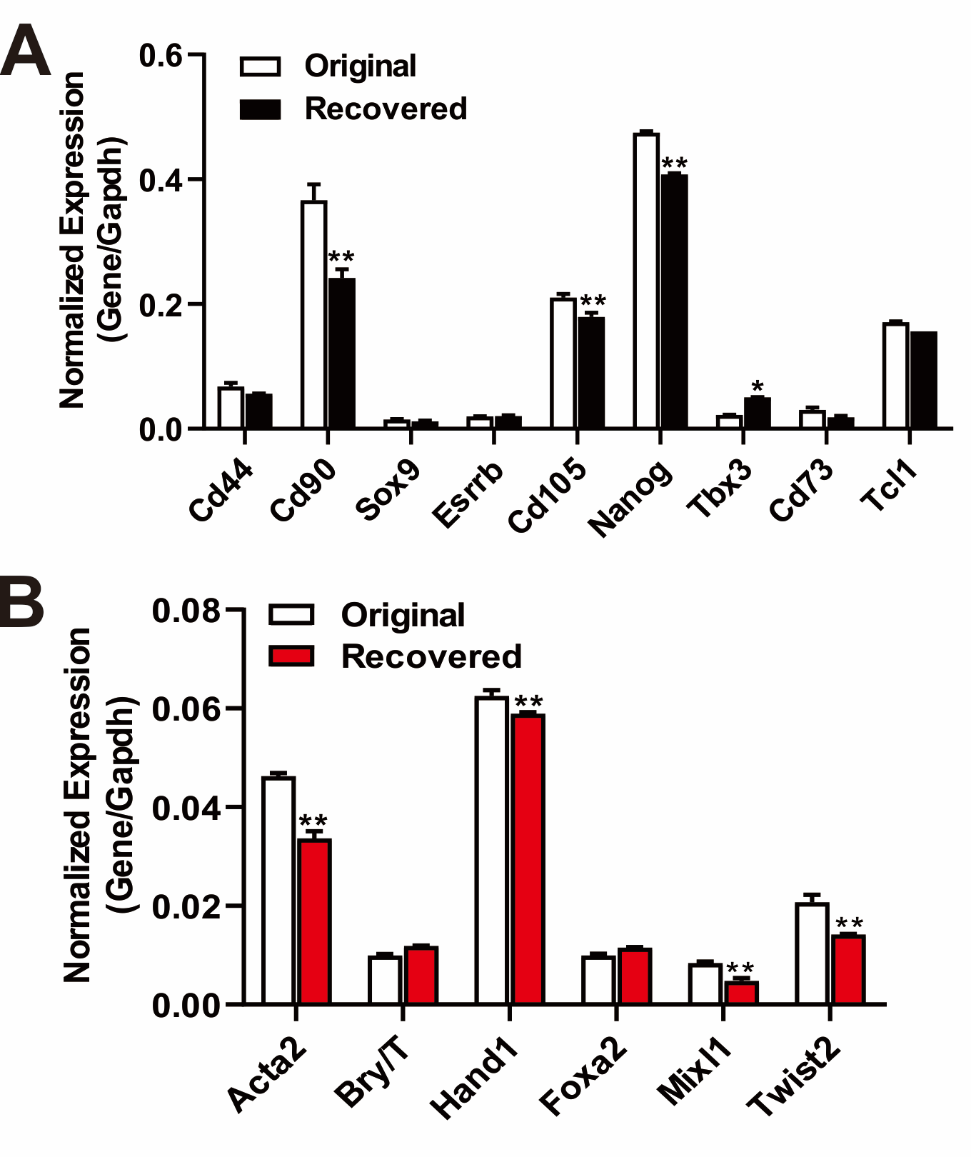


**Figure S6**. TqPCR analyses of the expression of stemness markers and mesoderm markers. 4 × 10^5^ iMEFs were suspended in 1 mL of 2% FBS DMEM in 1.5 mL sterile Eppendorf tubes at 16°C for 10 days. The original and recovered iMEFs were plated in 100 mm cell culture dishes and collected for RNA isolation at 24 h. TqPCR analysis was carried out to assess the expression of stemness markers **(A)** and mesoderm markers **(B)**. *Gapdh* was used as the reference gene. “**” *P* < 0.01, “*” *P* < 0.05, original iMEFs vs recovered iMEFs. The results are shown as heatmaps in **Fig. 1C, panels *c* & *d***.

**
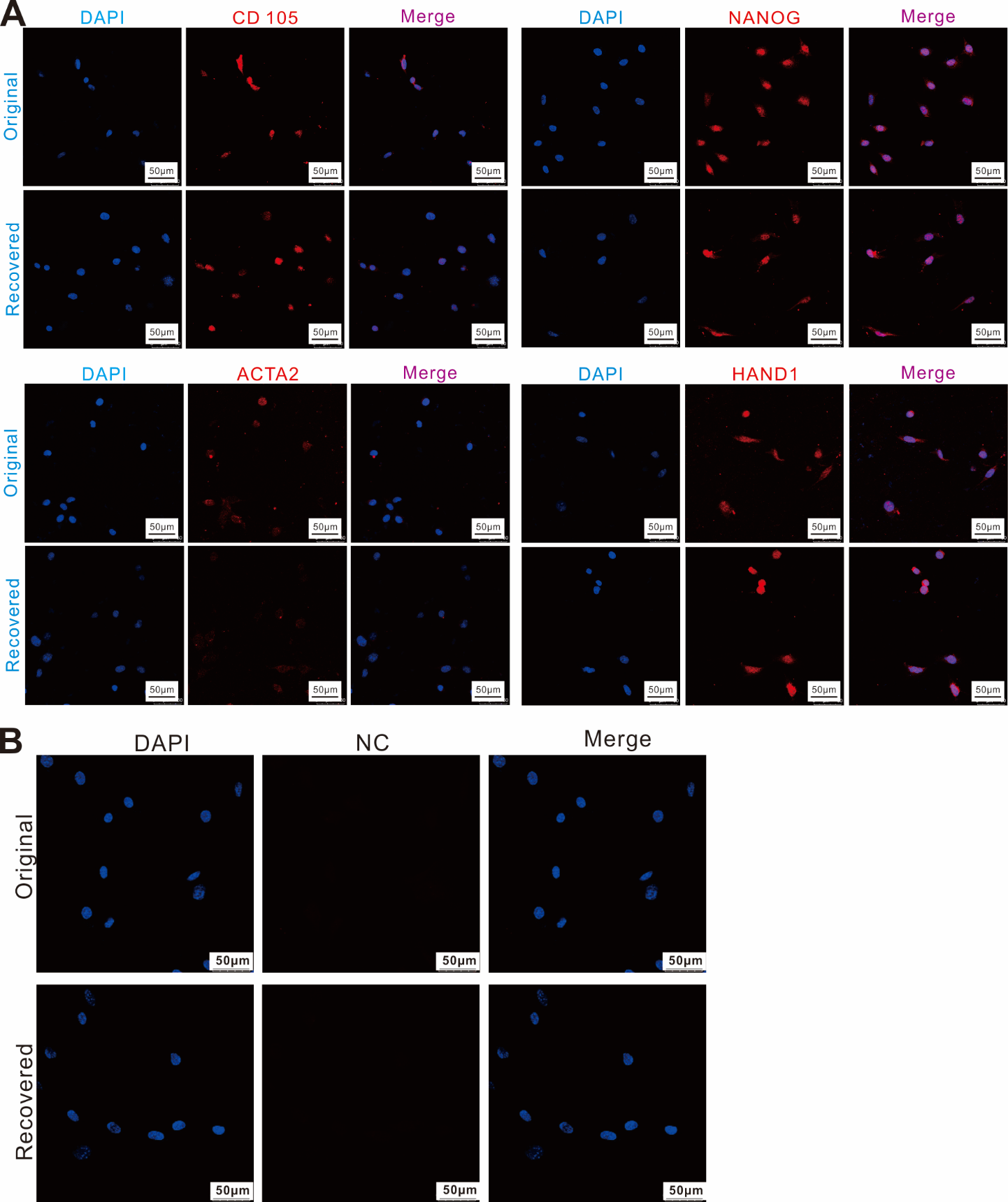
**

**Figure S7**. Immunofluorescence (IF) staining of MSC markers. 4 × 10^5^ iMEFs were suspended in 1 mL of 2% FBS DMEM in 1.5 mL sterile Eppendorf tubes at 16°C for 10 days. The original iMEFs and recovered iMEFs were seeded in 24-well cell culture plates, fixed, permeabilized and stained with primary antibodies against CD105, NANOG, ACTA2, and HAND1 (**A**) or the secondary antibody without the incubation with any primary antibodies (**B**). Cell nuclei were counter-stained with DAPI. Representative images are shown. Selected results are presented in **Fig. 1C panel *e***.


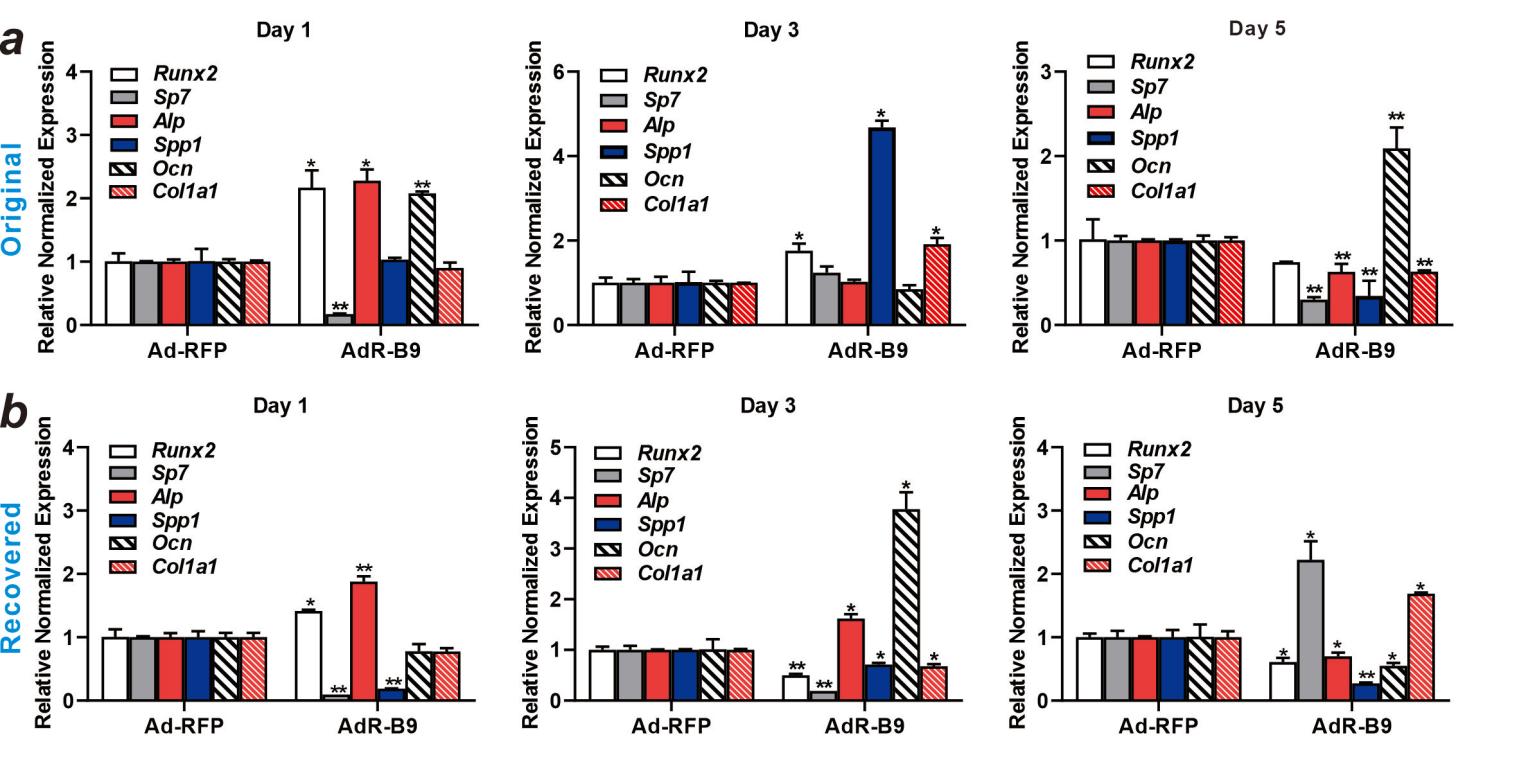


**Figure S8**. **TqPCR analysis of the expression of osteogenic markers**. 4 × 10^5^ iMEFs were suspended in 1 mL of 2% FBS DMEM in 1.5 mL sterile Eppendorf tubes and kept at 16°C for 10 days. The original (***a***) and recovered (***b***) iMEFs were seeded in 100 mm cell culture dishes and infected with AdR-B9 or Ad-RFP. Total RNA was collected at days 1, 3, and 5, and subjected to TqPCR analysis of the expression of *Runx2, Sp7, Alp, Spp1, Ocn,* and *Col1a1*. *Gapdh* was used as the reference gene. “**” *P* < 0.01, “*” *P* < 0.05, the original iMEFs vs. the recovered iMEFs at the indicated time points. The results are shown as a heatmap in **Fig. 1D panel *d***.


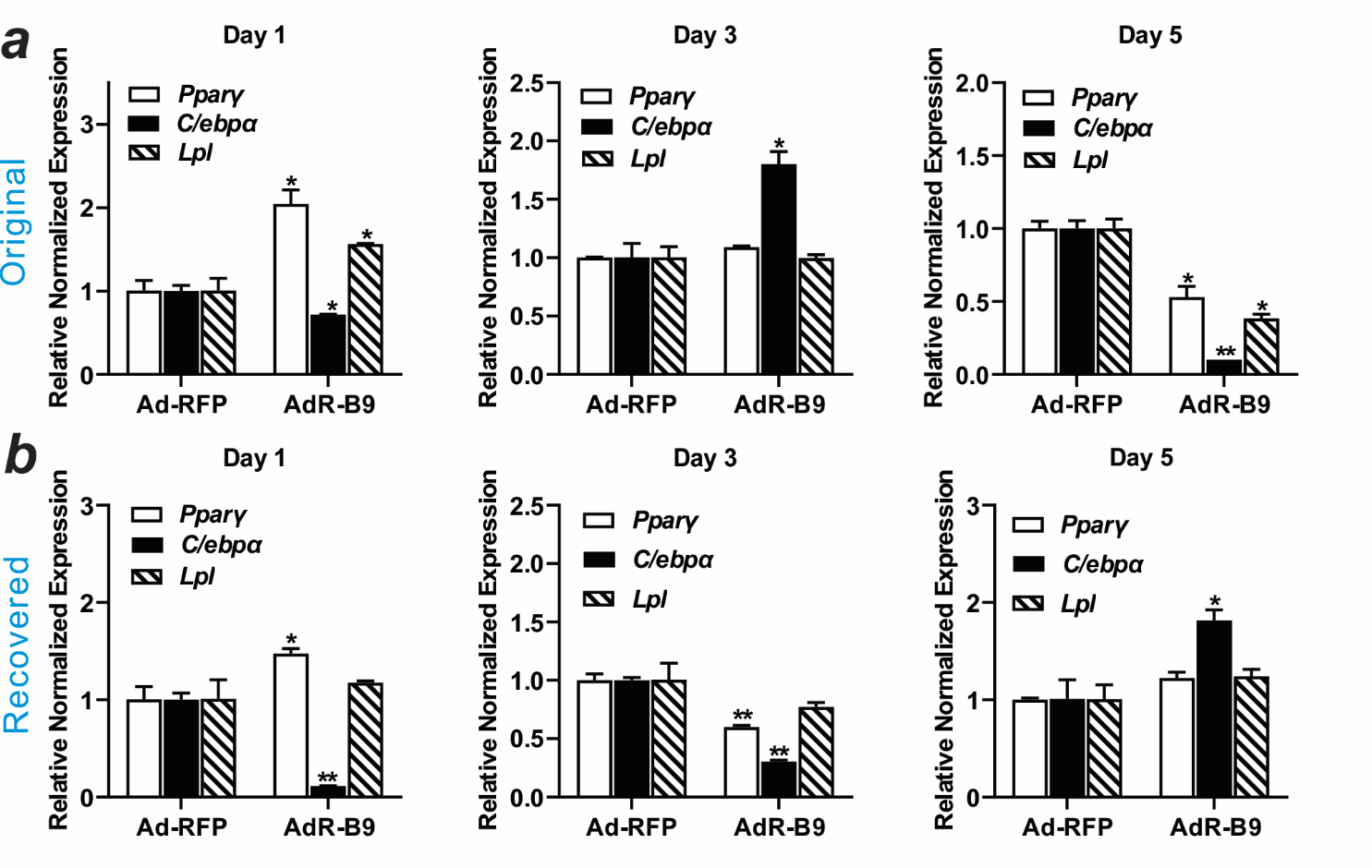


**Figure S9**. **TqPCR analysis of the expression of adipogenic markers**. 4 × 10^5^ iMEFs were suspended in 1 mL of 2% FBS DMEM in 1.5 mL sterile Eppendorf tubes and kept at 16°C for 10 days. The original (***a***) and the recovered (***b***) iMEFs were seeded in 100 mm cell culture dishes and infected with AdR-B9 or Ad-RFP. Total RNA was isolated at days 1, 3, and 5, and subjected to TqPCR analysis of the expression of *Pparγ*, *C/ebpα*, and *Lpl*. *Gapdh* was used as the reference gene. “**” *P* < 0.01, “*” *P* < 0.05, original iMEFs vs. the recovered iMEFs at indicated time points. The results are shown as a heatmap in **Fig. 1E panel *b***.


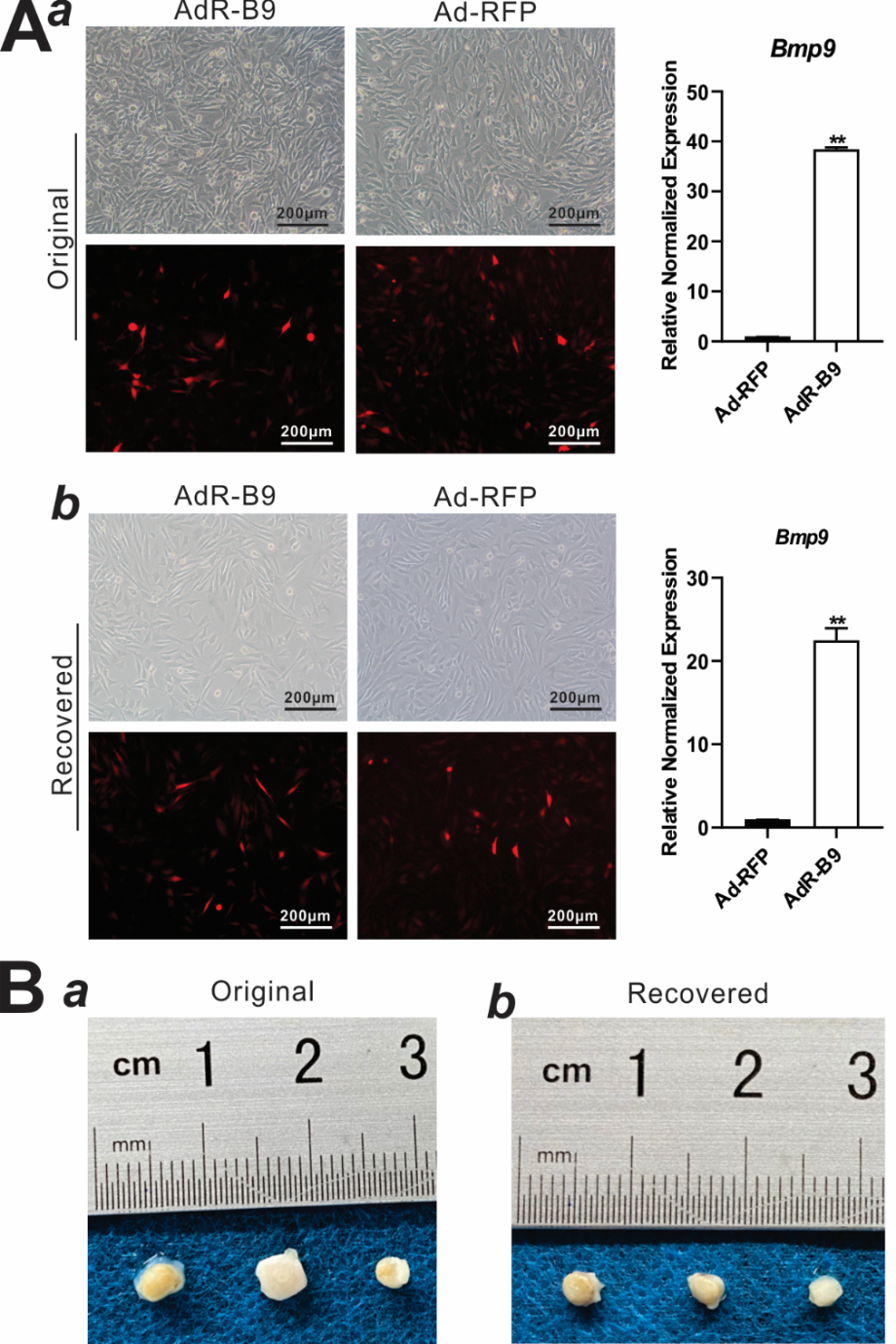


**Figure S10.** **(A)** The verification of AdR-B9 mediated overexpression of BMP9 in iMEFs for *in vivo* study**.** The original (***a***) and recovered (stored in 2% FBS DMEM at 16°C for 10 days) (***b***) iMEFs were infected with AdR-B9 or Ad-RFP, respectively, and fluorescence images were taken at 36 h after infection. TqPCR analysis was used to evaluate the expression of *Bmp9* at 48 h. “**” *p* < 0.01, AdR-B9 group vs Ad-RFP group. **(B)** Macrographic images of subcutaneous ectopic bone masses retrieved from AdR-B9 infected original iMEFs (***a***) or the recovered iMEFs stored in 2%FBS DMEM at 16°C for 10 days (***b***). No masses were retrieved from the Ad-RFP infected iMEFs in both groups. Representative images are shown.

**References**

1. Huang E, Bi Y, Jiang W, et al. Conditionally immortalized mouse embryonic fibroblasts retain proliferative activity without compromising multipotent differentiation potential. *PLoS One.* 2012;7(2):e32428.

2. Wang N, Zhang W, Cui J, et al. The piggyBac transposon-mediated expression of SV40 T antigen efficiently immortalizes mouse embryonic fibroblasts (MEFs). *PLoS One.* 2014;9(5):e97316.

3. Hu X, Li L, Yu X, et al. CRISPR/Cas9-mediated reversibly immortalized mouse bone marrow stromal stem cells (BMSCs) retain multipotent features of mesenchymal stem cells (MSCs). *Oncotarget.* 2017;8(67):111847-111865.

4. Wu N, Zhang H, Deng F, et al. Overexpression of Ad5 precursor terminal protein accelerates recombinant adenovirus packaging and amplification in HEK-293 packaging cells. *Gene Therapy.* 2014;21(7):629-637.

5. Yu Y, Zhong J, Chen C, et al. SV40 large T antigen-induced immortalization reprograms mouse cardiomyocyte progenitors with mesenchymal stem cell characteristics and osteogenic potential. *Genes & Diseases.* 2022. [https://doi.org/10.1016/j.gendis.2022.10.008](https://doi.org/10.1016/j.gendis.2022.10.008)

6. Wu X, Li Z, Zhang H, et al. Modeling colorectal tumorigenesis using the organoids derived from conditionally immortalized mouse intestinal crypt cells (ciMICs). *Genes Dis.* 2021;8(6):814-826.

7. Mao Y, Ni N, Huang L, et al. Argonaute (AGO) proteins play an essential role in mediating BMP9-induced osteogenic signaling in mesenchymal stem cells (MSCs). *Genes Dis.* 2021;8(6):918-930.

8. Huang L, Zhang J, Deng Y, et al. Niclosamide (NA) overcomes cisplatin resistance in human ovarian cancer. *Genes & Diseases.* 2023. [https://doi.org/10.1016/j.gendis.2022.12.005](https://doi.org/10.1016/j.gendis.2022.12.005)

9. Jiang B, Yan L, Miao Z, Li E, Wong KH, Xu RH. Spheroidal formation preserves human stem cells for prolonged time under ambient conditions for facile storage and transportation. *Biomaterials.* 2017;133:275-286.

10. Moritani Y, Usui M, Sano K, et al. Spheroid culture enhances osteogenic potential of periodontal ligament mesenchymal stem cells. *J Periodontal Res.* 2018;53(5):870-882.

11. Zhu Y, Shi Q, Peng Q, et al. A simplified 3D liver microsphere tissue culture model for hepatic cell signaling and drug-induced hepatotoxicity studies. *Int J Mol Med.* 2019;44(5):1653-1666.

12. Schwartz Z, Zhao P, Wang A, et al. Engineered nucleus-free mesenchymal stem cells (MSCs) for the targeted delivery of therapeutics to disease site. *Genes & Diseases.* 2022. [https://doi.org/10.1016/j.gendis.2022.09.001](https://doi.org/10.1016/j.gendis.2022.09.001)

13. Zhong J, Kang Q, Cao Y, et al. BMP4 augments the survival of hepatocellular carcinoma (HCC) cells under hypoxia and hypoglycemia conditions by promoting the glycolysis pathway. *Am J Cancer Res.* 2021;11(3):793-811.

14. Zhong J, Wang H, Yang K, et al. Reversibly immortalized keratinocytes (iKera) facilitate re-epithelization and skin wound healing: Potential applications in cell-based skin tissue engineering. *Bioact Mater.* 2022;9:523-540.

15. Kang Q, Song WX, Luo Q, et al. A comprehensive analysis of the dual roles of BMPs in regulating adipogenic and osteogenic differentiation of mesenchymal progenitor cells. *Stem Cells Dev.* 2009;18(4):545-559.

16. Cao D, Lei Y, Ye Z, et al. Blockade of IGF/IGF-1R signaling axis with soluble IGF-1R mutants suppresses the cell proliferation and tumor growth of human osteosarcoma. *Am J Cancer Res.* 2020;10(10):3248-3266.

17. Liu Q, Wang Z, Jiang Y, et al. Single-cell landscape analysis reveals distinct regression trajectories and novel prognostic biomarkers in primary neuroblastoma. *Genes Dis.* 2022;9(6):1624-1638.

18. Sun Y, He Y, Tong J, et al. All-trans retinoic acid inhibits the malignant behaviors of hepatocarcinoma cells by regulating ferroptosis. *Genes Dis.* 2022;9(6):1742-1756.

19. Zhang Q, Wang J, Deng F, et al. TqPCR: A Touchdown qPCR Assay with Significantly Improved Detection Sensitivity and Amplification Efficiency of SYBR Green qPCR. *PLoS One.* 2015;10(7):e0132666.

20. Fan J, Wei Q, Liao J, et al. Noncanonical Wnt signaling plays an important role in modulating canonical Wnt-regulated stemness, proliferation and terminal differentiation of hepatic progenitors. *Oncotarget.* 2017;8(16):27105-27119.

21. He TC, Zhou S, da Costa LT, Yu J, Kinzler KW, Vogelstein B. A simplified system for generating recombinant adenoviruses. *Proc Natl Acad Sci U S A.* 1998;95(5):2509-2514.

22. Luo J, Deng ZL, Luo X, et al. A protocol for rapid generation of recombinant adenoviruses using the AdEasy system. *Nat Protoc.* 2007;2(5):1236-1247.

23. Lee CS, Bishop ES, Zhang R, et al. Adenovirus-Mediated Gene Delivery: Potential Applications for Gene and Cell-Based Therapies in the New Era of Personalized Medicine. *Genes Dis.* 2017;4(2):43-63.

24. Yan S, Zhang R, Wu K, et al. Characterization of the essential role of bone morphogenetic protein 9 (BMP9) in osteogenic differentiation of mesenchymal stem cells (MSCs) through RNA interference. *Genes Dis.* 2018;5(2):172-184.

25. Zhao C, Wu N, Deng F, et al. Adenovirus-mediated gene transfer in mesenchymal stem cells can be significantly enhanced by the cationic polymer polybrene. *PLoS One.* 2014;9(3):e92908.

26. Zhang B, Yang L, Zeng Z, et al. Leptin Potentiates BMP9-Induced Osteogenic Differentiation of Mesenchymal Stem Cells Through the Activation of JAK/STAT Signaling. *Stem Cells Dev.* 2020;29(8):498-510.

27. Cheng H, Jiang W, Phillips FM, et al. Osteogenic activity of the fourteen types of human bone morphogenetic proteins (BMPs). *J Bone Joint Surg Am.* 2003;85(8):1544-1552.

28. Liao J, Wei Q, Zou Y, et al. Notch Signaling Augments BMP9-Induced Bone Formation by Promoting the Osteogenesis-Angiogenesis Coupling Process in Mesenchymal Stem Cells (MSCs). *Cell Physiol Biochem.* 2017;41(5):1905-1923.

29. Kang Q, Sun MH, Cheng H, et al. Characterization of the distinct orthotopic bone-forming activity of 14 BMPs using recombinant adenovirus-mediated gene delivery. *Gene Therapy.* 2004;11(17):1312-1320.

30. Wang H, Cao Y, Shu L, et al. Long non-coding RNA (lncRNA) H19 induces hepatic steatosis through activating MLXIPL and mTORC1 networks in hepatocytes. *J Cell Mol Med.* 2020;24(2):1399-1412.

31. Gou Y, Weng Y, Chen Q, et al. Carboxymethyl chitosan prolongs adenovirus-mediated expression of IL-10 and ameliorates hepatic fibrosis in a mouse model. *Bioeng Transl Med.* 2022;7(3):e10306.

32. He F, Ni N, Zeng Z, et al. FAMSi: A Synthetic Biology Approach to the Fast Assembly of Multiplex siRNAs for Silencing Gene Expression in Mammalian Cells. *Mol Ther Nucleic Acids.* 2020;22:885-899.

33. Ni N, Deng F, He F, et al. A one-step construction of adenovirus (OSCA) system using the Gibson DNA Assembly technology. *Mol Ther Oncolytics.* 2021;23:602-611.
